# Identification of Differentially Expressed Genes and Prediction of Expression Regulation Networks in Dysfunctional Endothelium

**DOI:** 10.3390/genes13091563

**Published:** 2022-08-30

**Authors:** Fang Cheng, Yujie Zeng, Minzhu Zhao, Ying Zhu, Jianbo Li, Renkuan Tang

**Affiliations:** 1 Department of Forensic Medicine, Faculty of Basic Medical Science, Chongqing Medical University, Chongqing 400016, China; 2 Chongqing Engineering Research Center for Criminal Investigation Technology, Chongqing 400016, China; 3 Chongqing Key Laboratory of Forensic Medicine, Chongqing 400016, China

**Keywords:** early coronary atherosclerosis, endothelial dysfunction, TOX, RasGRP3, TSPAN13

## Abstract

The detection of early coronary atherosclerosis (ECA) is still a challenge and the mechanism of endothelial dysfunction remains unclear. In the present study, we aimed to identify differentially expressed genes (DEGs) and the regulatory network of miRNAs as well as TFs in dysfunctional endothelium to elucidate the possible pathogenesis of ECA and find new potential markers. The GSE132651 data set of the GEO database was used for the bioinformatic analysis. Principal component analysis (PCA), the identification of DEGs, correlation analysis between significant DEGs, the prediction of regulatory networks of miRNA and transcription factors (TFs), the validation of the selected significant DEGs, and the receiver operating characteristic (ROC) curve analysis as well as area under the curve (AUC) values were performed. We identified ten genes with significantly upregulated signatures and thirteen genes with significantly downregulated signals. Following this, we found twenty-two miRNAs regulating two or more DEGs based on the miRNA–target gene regulatory network. TFs with targets ≥ 10 were E2F1, RBPJ, SSX3, MMS19, POU3F3, HOXB5, and KLF4. Finally, three significant DEGs (TOX, RasGRP3, TSPAN13) were selected to perform validation experiments. Our study identified TOX, RasGRP3, and TSPAN13 in dysfunctional endothelium and provided potential biomarkers as well as new insights into the possible molecular mechanisms of ECA.

## 1. Introduction

Coronary atherosclerosis (CA), as the basic pathological basis of coronary heart disease (CHD), is gradually becoming the leading cause of death all over the world, posing a serious threat to human life and health. CA progresses gradually and is usually asymptomatic at an early stage at which it can be effectively treated [1,2]. Therefore, the early diagnosis and intervention of CA are urgent [3].

Atherosclerosis results in the impairment of the structure and function of the intima, media, and adventitia of the coronary artery wall [4]. The intima is composed of the endothelium and the subendothelial layer, which is the thinnest of the three layers [5]. Studies on the fundamental mechanism of atherosclerosis have shown that endothelial dysfunction is an early vital point in the development of atherosclerosis and is also involved in the progression of plaques and the occurrence of atherosclerotic complications [6,7]. As the earliest detectable changes in atherosclerosis, endothelial dysfunction occurs in vulnerable areas of arterial vasculature [8]. Endothelial dysfunction, which is regarded as a constant imbalance of vasoactive substances, showing an unbalanced reaction between vasodilator and vasoconstrictor in response to endothelial-dependent vasodilating agents such as acetylcholine, causes the failure of the endothelium to control tissue perfusion [9,10]. Therefore, the detection of early coronary atherosclerosis (ECA) is critical for prognostics and prevention, as well as therapeutics and the delay of atherosclerosis progression. However, the mechanism of endothelial dysfunction remains unclear and the detection of ECA is still a challenge.

MicroRNAs (miRNAs), a class of non-protein coding RNA with a length from 18 to 25 nucleotides, act as a kind of regulator of mRNA expression via inducing mRNA degradation or inhibiting mRNA translation at the post-transcription level, causing the subsequent translation or non-translation of extensive target genes that contribute to a wide range of biological processes [11]. miRNAs have been associated with numerous cardiovascular diseases since their discovery in 1993 by Lee and colleagues [12]. For CA, various miRNAs have been found to be biomarkers in the diagnosis of CA and their functional role in primary cardiovascular prevention has been revealed [13]. However, whether miRNAs are involved in the occurrence and development of ECA and which target genes they regulate remain to be further studied. As a group of protein molecules, transcription factors (TFs) can be specifically bound to the ends of specific upstream sequence genes of 5′ so as to ensure that the target genes are expressed at a specific intensity in a specific time and space [14].

To study the regulation of gene expression, TF is generally regarded as a prerequisite and forms the basis. Therefore, it is also necessary to study the role of TFs in ECA or the signal pathways involved. The interaction between target genes, miRNAs, and TFs provides a vast diversity of possibilities for the regulation of health and disease. In this study, bioinformatics and IHC were used to analyze the relationship between endothelial gene expression and ECA and to predict the miRNAs and TFs that regulate these genes in an attempt to elucidate the possible molecular mechanism and find a potential marker for diagnosis.

## 2. Materials and Methods

### 2.1. Data Extraction Based on GEO Database

GEO (https://www.ncbi.nlm.nih.gov/geo/ (accessed on 15 April 2022)) database, an open database affiliated with the National Center for Biotechnology Information (NCBI), contains high-throughput gene expression data, gene microarray, gene chip, and other gene expression abundance information. The gene chip data set related to early coronary atherosclerosis was searched in GEO, and the GSE132651 data set was retrieved and downloaded in an R environment using the GEOquery package [15]. The GSE132651 data set included 6 samples of normal coronary artery endothelial function and 13 samples of abnormal coronary artery endothelial function, all of which were adults under 50 years old. Abnormal coronary artery endothelial function, namely coronary endothelial dysfunction, serves as the earliest clinically detectable form of an atherosclerotic lesion, providing gold-standard evidence for the existence of atheropromotive pathobiology [16].

### 2.2. Differential Gene Screening and Data Processing

We used FactoMineR and the factoextra package in R software (R Foundation for Statistical Computing, Vienna, Austria) to conduct a principal component analysis (PCA) on the two groups of samples in the data set and make a diagram. Differentially expressed genes (DEGs) were obtained and identified from samples with abnormal coronary artery endothelial function and normal samples by the limma package in R software, and the significant, differentially expressed upregulated or downregulated genes were obtained under the screening conditions of *p* < 0.05, log2FC (fold change) > 1 or log2FC < −1. The correlation map, volcano map, and heatmap of significant DEGs were drawn by package corrplot, ggplot2, and pheatmap in R software, respectively.

### 2.3. Construction of the miRNA–Target Gene and Target Gene Transcription Factor (TF) Regulatory Network

miRNAs and TFs are two types of trans-regulators that can modulate gene regulatory networks in a dependent or independent manner [17]. Thus, the context of the regulatory interactions of TFs and miRNAs may help to understand the function of DEGs deeply. For predicting miRNA regulating the DEGs, we used FunRich software (Mathivanan Lab of La Trobe University, Sydney, Australia) and then input the data into Cytoscape to build a miRNA–target gene regulatory network. Potential TFs in relation to the DEGs were predicted utilizing iRegulon [18] with enrichment score threshold = 5.0, ROC threshold for AUC calculation = 0.05, rank threshold = 5000, minimum identity between orthologous genes = 0.05, and maximum FDR on motif similarity = 0.001 in Cytoscape, and the target gene–TF regulatory networks were constructed.

### 2.4. Immunohistochemistry Validation

In order to confirm the reliability of significant DEGs, three significant DEGs, thymocyte selection associated high mobility group box (TOX), RAS guanyl-releasing protein 3 (RasGRP3), and tetraspanin 13 (TSPAN13) were selected for validation by IHC analyses. All coronary artery samples were collected from forensic autopsy cases of 15 cadavers aged from 22 to 57 years from 2020 to 2021, and the procedure was approved by the ethical committee of Chongqing Medical University. Table 1 summarized the main characteristics of these samples. The proximal sections of the left anterior descending artery (LAD) were collected, dissected from fat and connective tissue, and immersed into 10% formalin. According to the extent of coronary atherosclerotic plaque protruding into the lumen in the HE staining results, the samples were divided into a non-coronary artery stenosis group (group A) and a coronary artery stenosis <25% group (group B). The primary antibodies included monoclonal anti-rabbit antibody to the protein TOX (dilution 1:1000; #73758, Cell Signaling Technology, Danvers, MA, USA), polyclonal anti-rabbit antibody to the protein RasGRP3 (dilution 1:100; D227459, Sangon Biotech, Shanghai, China) and TSPAN13 (dilution 1:50; D262375, Sangon Biotech). Negative control sections were incubated without the primary antibody. Five different fields of each formalin-fixed LAD tissue section were randomly selected by two independent investigators to evaluate the IHC staining. The evaluation of IHC staining results was operated by a semi-quantitative classification system, as described by Mayer et al. [19]: score 0, no positive staining; score 1, positive staining of single cells; score 2, positive staining of groups of cells; score 3, positive staining of cells in large tissue areas. The receiver operating characteristic (ROC) curve analysis and area under the curve (AUC) values were performed by the pROC and ggplot2 packages in R software.

### 2.5. Statistical analysis

Statistical analysis was performed using SPSS (version 20.0, SPSS Company, Chicago, IL, USA) and GraphPad Prism (version 8.0.1, La Jolla, CA, USA) software. The Mann–Whitney U test was utilized for the comparison of two sample groups. Differences were considered statistically significant when *p* < 0.05.

## 3. Results

### 3.1. Identification and Correlation Analysis of Significant DEGs in ECA

**Figure 1:**
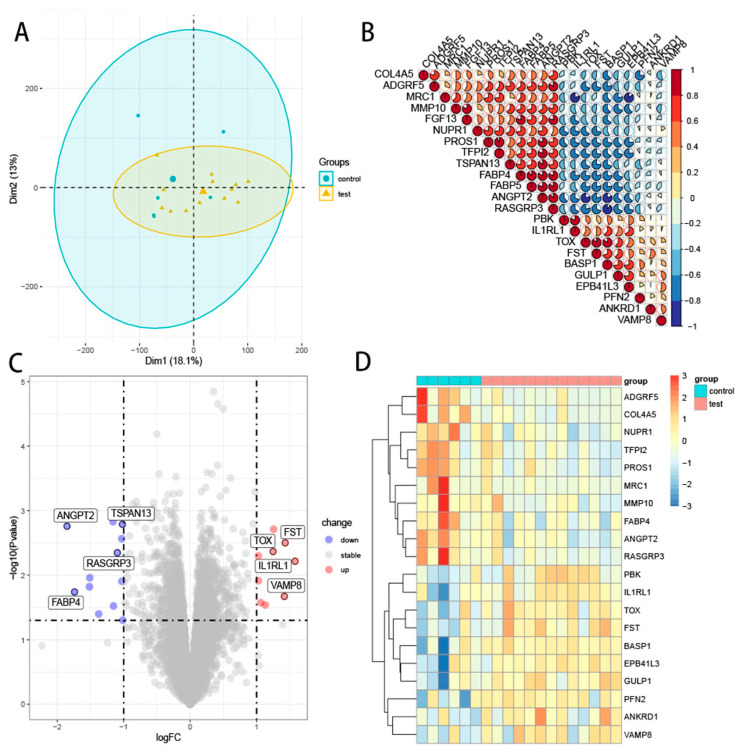
Identification and correlation analysis of significant DEGs in ECA. (**A**) PCA of the two groups. Each point in the graph represents a sample, and the distance between points represents the difference between samples. (**B**) Gene correlation analysis of 23 significant DEGs. Red to blue indicates a strong to weak correlation. (**C**) Volcano plot of the significant DEGs in the two groups. The screening conditions were *p* < 0.05 and |log_2_FC| > 1. Red represents high expression and green represents low expression. Grey shows no differential expression. (**D**) Heat map of significant DEGs in each sample. Red to blue indicates a strong to weak correlation. (DEGs, differentially expressed genes; ECA, early coronary atherosclerosis; PCA, principal component analysis).

In order to examine whether the two groups of samples cluster separately, we used PCA, and the result showed two clearly separated clusters for the control and test samples, which indicated that there was a difference between the two groups (Figure 1A). Then, setting *p* < 0.05, |log2FC| > 1 as screening conditions, ten genes were significantly upregulated and thirteen genes were remarkably downregulated (Table 2). The twenty-three significant DEGs were analyzed for gene correlation, as shown in Figure 1B. The expression of significant DEGs in the two groups and each sample is shown in Figure 1C,D, respectively.

### 3.2. Construction of the miRNA–Target Gene and Target Gene–TF Regulatory Network

**Figure 2:**
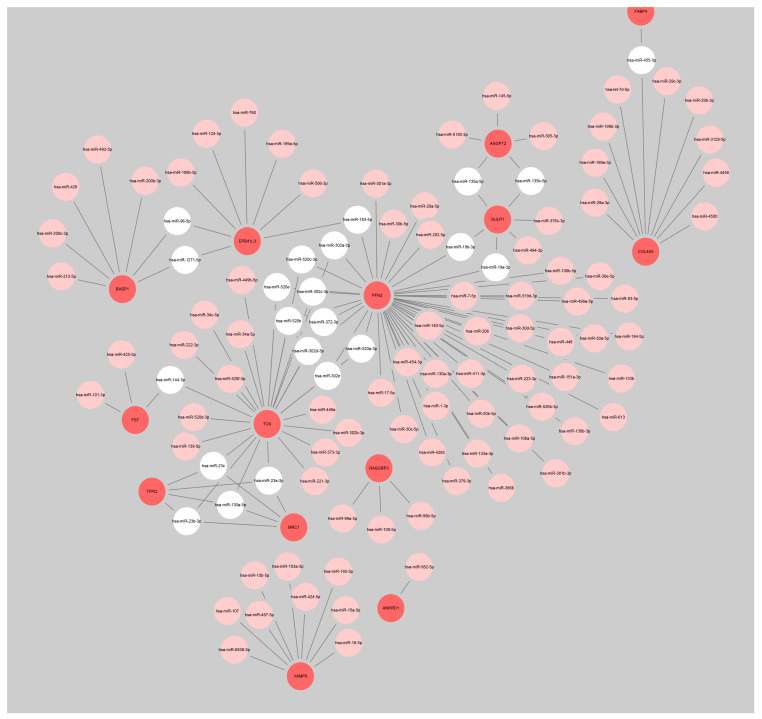
Network diagram of miRNAs regulating 23 significant DEGs. Bright red represents 23 significant DEGs, pink represents miRNAs, and white represents miRNAs that simultaneously regulate two or more significant DEGs.

**Figure 3:**
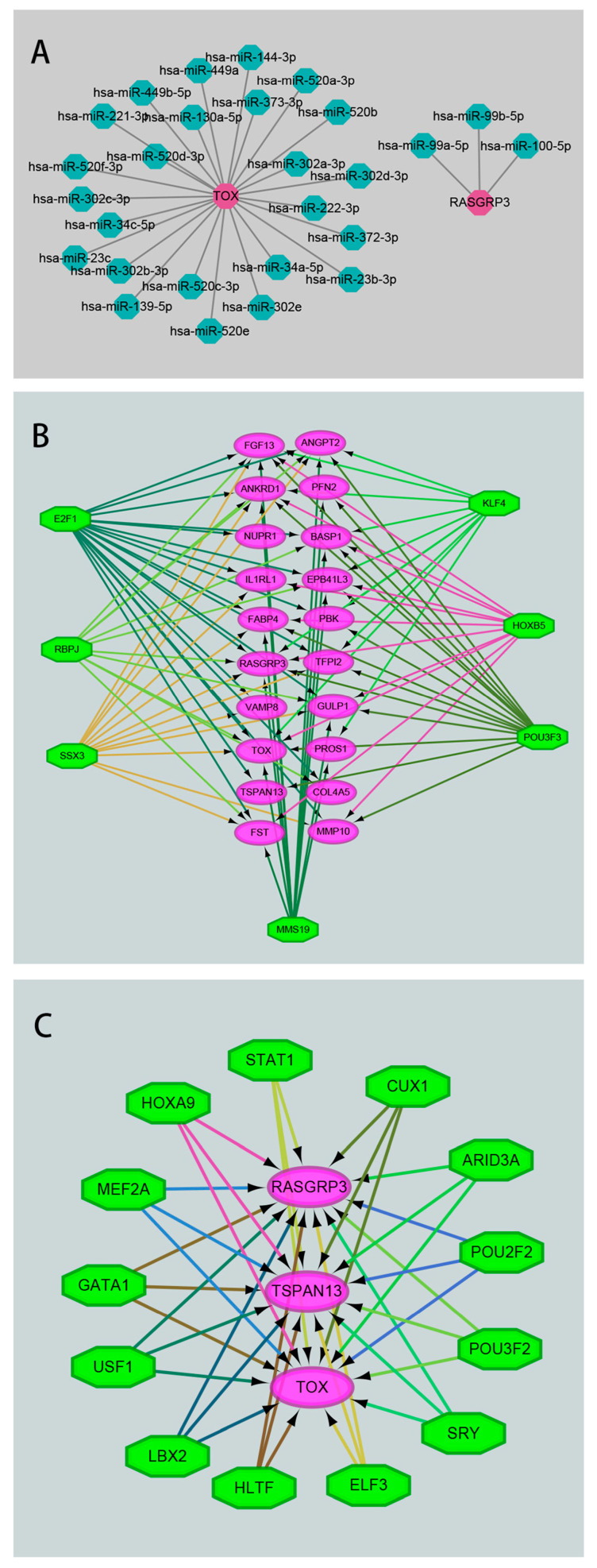
The miRNA–target gene and target gene–TF regulatory network. (**A**) Network diagram of miRNAs regulating TOX or RasGRP3. Bright red represents TOX or RasGRP3, green represents miRNAs. (**B**) Network diagram of TFs regulating 23 significant DEGs. Purple circles indicate 23 significant DEGs, green octagons indicate TFs with targets ≥ 10. (**C**) Network diagram of TFs simultaneously regulating TOX, RasGRP3, and TSPAN13. Purple circles indicate TOX, RasGRP3, or TSPAN13, green octagons indicate TFs.

Given the possible role of miRNA and TF in regulating the expression of genes, we further investigated miRNAs that may regulate these significant DEGs. As shown in Figure 2, there were twenty-two miRNAs regulating two or more DEGs, namely hsa-miR-455-3p, hsa-miR-135b-5p, hsa-miR-135a-5p, hsa-miR-19a-3p, hsa-miR-19b-3p, hsa-miR-183-5p, hsa-miR-302a-3p, hsa-miR-520c-3p, hsa-miR-302c-3p, hsa-miR-372-3p, hsa-miR-520a-3p, hsa-miR-302e, hsa-miR-302d-3p, hsa-miR-520b, hsa-miR-520e, hsa-miR-96-5p, hsa-miR-1271-5p, hsa-miR-144-3p, hsa-miR-23a-3p, hsa-miR-130a-5p, hsa-miR-23c, and hsa-miR-23b-3p. Among the three significant DEGs we were most concerned about, it is worth noting that miRNAs regulating TSPAN13 had not been predicted, while miRNAs regulating TOX and RasGRP3 are shown in Figure 3A. Next, the TFs that regulate these significant DEGs were predicted. TFs with target ≥ 10 were E2F1, RBPJ, SSX3, MMS19, POU3F3, HOXB5, and KLF4 (Figure 3B). TFs that can regulate all the three significant DEGs of TOX, TSPAN13, and RasGRP3 were STAT1, CUX1, ARID3A, POU2F2, POU3F2, SRY, ELF3, HLTF, LBX2, USF1, GATA1, MEF2A, and HOXA9 (Figure 3C). 

### 3.3. Validation of the Microarray Data by IHC

**Figure 4:**
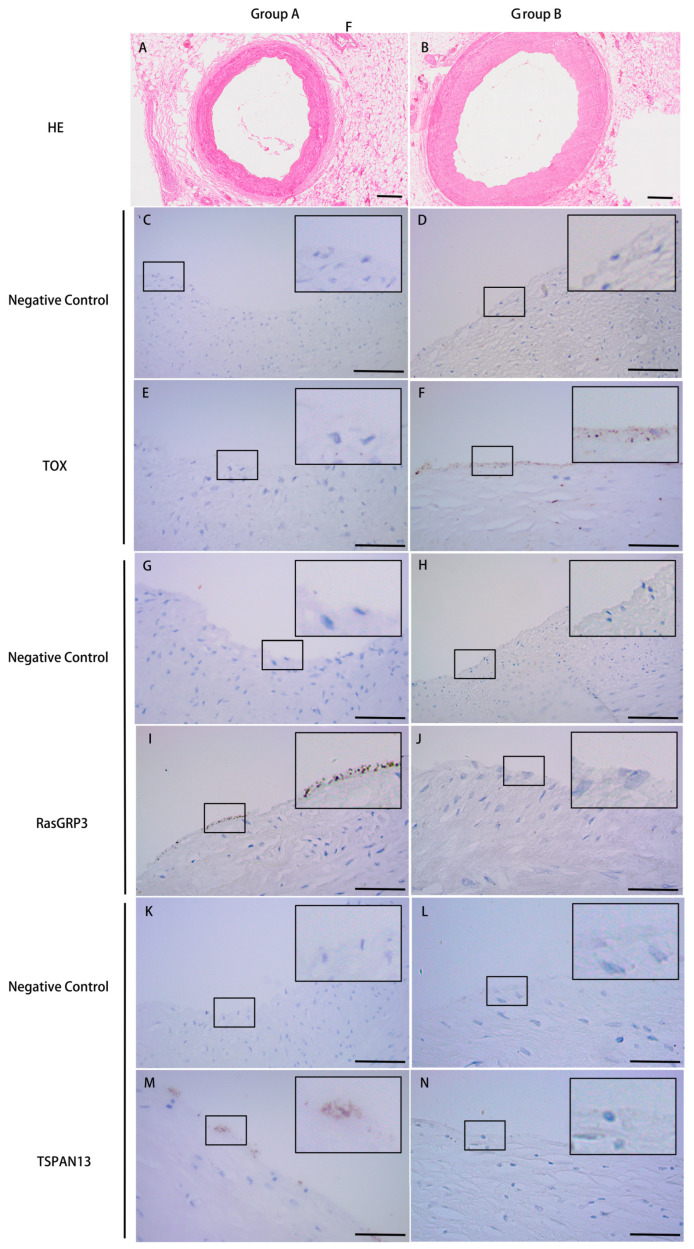
HE staining and validation of the microarray data by IHC. (**A**) Representative figures of HE staining in group A without coronary stenosis. (**B**) Representative figures of HE staining in group B with coronary stenosis less than 25%. (**C**) Negative control of TOX in group A. (**D**) Negative control of TOX in group B. (**E**) Representative IHC figures of TOX in group A. (**F**) Representative IHC figures of TOX in group B. Compared to group A, the expression of TOX in group B was significantly upregulated (*p* = 0.031). (**G**) Negative control of RasGRP3 in group A. (**H**) Negative control of RasGRP3 in group B. (**I**) Representative IHC figures of RasGRP3 in group A. (**J**) Representative IHC figures of RasGRP3 in group B. Compared to group A, the expression of RasGRP3 in group B was significantly downregulated (*p* = 0.032). (**K**) Negative control of TSPAN13 in group A. (**L**) Negative control of TSPAN13 in group B. (**M**) Representative IHC figures of TSPAN13 in group A. (**N**) Representative IHC figures of TSPAN13 in group B. Compared to group A, the expression of TSPAN13 in group B was significantly downregulated (*p* = 0.046). Magnification of HE: 200×. Magnification of IHC: 400×. bar = 50 μm.

**Figure 5:**
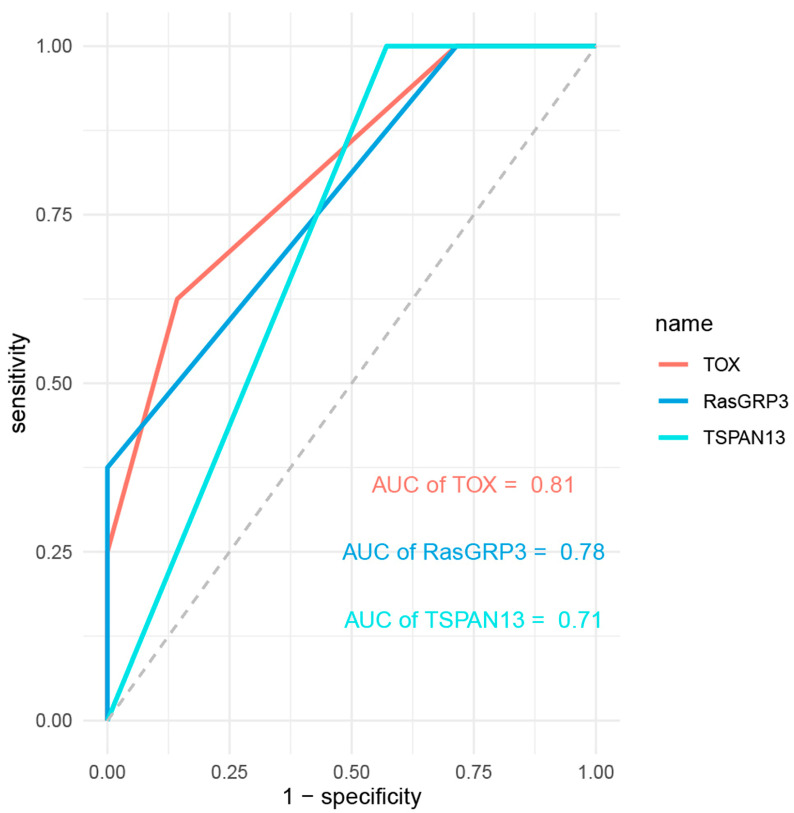
ROC figure of TOX, RasGRP3, and TSPAN13. The AUC of TOX, RasGRP3, and TSPAN13 was 0.81, 0.78, and 0.71, respectively.

Three significant DEGs—TOX, RasGRP3, and TSPAN13—were selected for IHC analysis based on their high fold changes and possible biological functions in the development of early coronary atherosclerosis to validate the bioinformatics analysis results. Representative figures of HE staining in groups are shown in Figure 4A,B. Figure 4C,D,G,H,K,L shows the negative controls for the TOX, RasGRP3, and TSPAN13 of group A and group B, respectively. Compared with group A, the verification results confirmed that the expression of TOX (Figure 4E,F) was upregulated, RasGRP3 (Figure 4I,J) and TSPAN13 (Figure 4M,N) were downregulated in group B, and the differences were statistically significant (*p* = 0.031, *p* = 0.032 and *p* = 0.046, respectively). The expression of TOX, RasGRP3, and TSPAN13 showed no significant correlation with age, sex, PMI, and cause of death in group A and group B. The above results were consistent with the bioinformatics analysis results, which further supported the accuracy and reliability of the present analysis. Based on the ROC curve analysis, the AUC of TOX, RasGRP3, and TSPAN13 was 0.81, 0.78, and 0.71, respectively (Figure 5). 

## 4. Discussion

The high incidence and mortality of CHD are cardinal concerns worldwide. CHD not only impairs individual health but also imposes heavy financial burdens on countries. In the early stage of CHD, the diagnosis of ECA is conducive to taking measures to intervene in its progress promptly. Therefore, it is of vital importance to figure out the potential mechanisms and biomarkers of ECA. In the current study, we used bioinformatical methods to identify DEGs for ECA based on the gene expression profile dataset (GSE132651) and to reveal the molecular mechanisms of coronary endothelial dysfunction in CHD. We found that 10 genes were significantly upregulated and 13 genes were significantly downregulated (*p* < 0.05, |log2FC| > 1) via R software. Next, the miRNAs and TFs that regulate these significant DEGs were predicted using Funrich and Cytoscape software, respectively, and the miRNA–target gene and target gene–TF regulatory networks were constructed. Three significant DEGs (TOX, RasGRP3, and TSPAN13) were selected for IHC analysis and the findings were consistent with the bioinformatics analysis results. The identification and analysis of ECA-associated genes, miRNAs, and TFs may reveal the potential pathogenesis of ECA on the level of molecules and help to facilitate ECA diagnosis and treatment.

TOX (also known as KIAA0808), a highly conserved transcription factor, belongs to the superfamily of high mobility group box (HMG-box) proteins and is expressed in many human tissues, including the thymus, liver, brain, spleen, bone marrow, lung, kidney, and breast [20,21,22]. A couple of studies have reported that TOX is crucial to the immune system, such as the development of T cells [23,24] and the formation of NK cells [25]. TOX is transiently elevated via TCR-mediated calcineurin signaling during mouse β selection and positive selection in the thymus and reduced with the maturation of CD4^+^ and CD8^+^ T cells [21,23]. Moreover, recent research has suggested that TOX serves as an important transcription factor in the process of CD8^+^ T cell exhaustion [26,27,28], which is characterized by the decreased cytokine production and cytolytic activity of CD8^+^ T cells. CD8^+^ T cells, a kind of immune cell, accumulate in the intima of arteries in atherosclerosis [29]. In vitro, the apoptosis of human endothelial cells could be induced by CD8^+^ T cells and their cytotoxic effector molecules, and the adhesion of pre-activated CD8^+^ T cells and endothelial cells was increased under disturbed flow conditions [30]. In addition, TOX is also implicated in chronic infection and cancer. TOX was a biomarker specific for dysfunctional virus-specific CD8^+^ T cells in the context of an actively persisting infection [31]. The expression of TOX is negatively correlated with DNA methylation in tumor cells and may serve as a novel prognostic marker for breast cancer and lung adenocarcinoma [22,32]. Our results showed that the expression of TOX was significantly increased in the coronary endothelium of ECA patients. Various clues suggested that TOX may be involved in atherosclerosis, but its role in ECA has not been reported.

RasGRP3, non-PKC DAG/phorbol ester receptors, is one of the guanine nucleotide exchange factors (GEFs) and activates the Ras family of GTPases [33,34]. RasGRP3 is mainly expressed in B cells, macrophages, and endothelial cells. A study of the murine-based gene-trap screen has reported that RasGRP3 was a locus expressed in the endothelial cells of developing vessels and is essential for mediating the endothelial cell impacts of DAG/phorbol esters [35]. In recent years, RasGRP3 also has been reported to play a vital functional role in cancer formation and progression [36]. For example, the expression of RasGRP3 was selectively and significantly increased in the uveal melanoma of GNAQ/11 mutation [37]. Additionally, the tissue RasGRP3 mRNA was remarkably elevated in mitral regurgitation patients with heart failure [38]. Due to RasGRP3 being expressed in endothelial cells and related to a variety of diseases, and the fact that this study found that the expression of RasGRP3 in endothelial cells of ECA was different from normal, we speculated that RasGRP3 was involved in coronary atherosclerosis, but this has not been reported so far.

TSPAN13 (also known as NET-6) belongs to a superfamily of small transmembrane proteins that are implicated in cell migration, proliferation, signal-transduction, intracellular trafficking, and virus infection [39]. TSPAN13 in breast cancer cells hinders growth and invasion, as well as elevating apoptosis in vitro and in vivo [40]. Moreover, TSPANs are extensively expressed in the cardiovascular system. It was found that the overexpression of TSPAN13 enhanced the formation of migrasomes, which function in regulating vascular homeostasis [41]. Of our results, the expression of TSPAN13 was significantly decreased in the coronary endothelium of ECA patients. All in all, there are limited studies on TSPAN13. Thus, further studies are needed to confirm the current findings and investigate the role of TSPAN13 in the endothelial cells of ECA.

The prediction of miRNA is one of the most essential fields in gene research all the time, given that miRNAs exert their function by regulating target mRNAs. According to our research, twenty-one miRNAs regulating TOX and three miRNAs regulating RasGRP3 were predicted, but no miRNA regulating TSPAN13 was predicted, which may be due to the lack of research on TSPAN13 at present. Some studies have reported that miRNAs are involved in the occurrence and development of coronary atherosclerosis, and even might be used as potential biomarkers. miRNAs can integrate their effects to accomplish a nuanced result, no matter whether the result is beneficial or detrimental, by controlling the expression of many targets and gene networks. Chang et al. identified eight exosomal miRNA profiles from exosomes extracted from coronary blood samples as potential biomarkers and predictors of stable CA [42]. Some miRNAs, such as miR-139-5p [43], miR-145 [44], miR-100 [45], miR-194-5p [46], miR-199a-3p [46] and miR-199b-3p [46], which may regulate CA, have been identified. Overall, the role of miRNAs in cardiovascular diseases has been widely described. Secondly, thirteen TFs that can regulate all three genes verified by IHC were shown in the network. We speculate that TFs, which can regulate all three genes, are more likely to participate in the mechanism of significant changes in their expression. Researchers have revealed that E2F1 [47], KLF4 [48,49], STAT1 [50], and HOXA9 [51] may be involved in the pathogenesis of CHD. We believe that these miRNAs and TFs may provide new insights for the further research of ECA.

In the current study, we have mainly discussed three significant DEGs (TOX, RasGRP3, TSPAN13) and miRNAs as well as the TFs that may regulate them in the endothelial cells of ECA based on the bioinformatics analysis of the GSE132651 data set and the IHC validation experiments of coronary artery samples collected from forensic autopsy cases. Yet, the limitations of our study should also be recognized. According to the results, the expression of the three DEGs was identified to have changed, but the mechanism of alteration remains unclear. Hence, further evidence is required to find out the biological foundation.

## 5. Conclusions

We mainly identified TOX, RasGRP3, and TSPAN13 in the dysfunctional endothelium of ECA in combination with the regulatory network of miRNAs and TFs by bioinformatics analysis as well as IHC validation experiments, suggesting that TOX, RasGRP3, and TSPAN13 may serve as potential biomarkers or factors for ECA. The findings of our study may provide new insights into the molecular mechanisms of ECA.

## Figures and Tables

**Table 1:** Detailed characteristics of the study collective for IHC validation.

Group	No.	Sex	Age	PMI	Cause of Death	Score of IHC Staining		
			(years)	(days)		TOX	RasGRP3	TSPAN13
A	1	M	30	11	poisoning	1	1	2
A	2	M	25	2	accident	0	1	1
A	3	F	34	4	cardiomyopathy	1	2	2
A	4	M	22	1	head injury	2	1	1
A	5	M	30	5	electric shock	1	1	1
A	6	M	26	4	pneumonia	1	1	2
A	7	M	37	3	head injury	0	2	1
B	8	M	46	1	head injury	1	1	1
B	9	M	56	23	cardiomyopathy	2	1	1
B	10	M	44	2	chest injury	1	0	1
B	11	M	57	3	pulmonary embolism	1	1	1
B	12	F	47	5	cardiomyopathy	2	0	1
B	13	M	52	1	liver cancer	3	0	1
B	14	M	29	8	alcoholism	2	1	1
B	15	M	49	8	rupture of spleen	3	1	1

M: male; F: Female; PMI: postmortem interval; 0: no positive staining; 1: positive staining of single cells; 2: positive staining of groups of cells; 3: positive staining of cells in large tissue areas.

**Table 2:** Twenty-three genes with significant differences in expression.

Probe_id	Gene Symbol	Log_2_FC	*p* Value	adj. *p* Value	*t*	B	Change
205572_at	ANGPT2	−1.853	0.002	0.363	−3.613	−1.228	down
204948_s_at	FST	1.430	0.003	0.379	3.356	−1.690	up
204529_s_at	TOX	1.248	0.004	0.391	3.221	−1.932	up
202391_at	BASP1	1.255	0.002	0.369	3.566	−1.314	up
213110_s_at	COL4A5	−1.160	0.001	0.363	−3.683	−1.103	down
204235_s_at	GULP1	1.084	0.003	0.372	3.413	−1.589	up
205801_s_at	RASGRP3	−1.104	0.004	0.391	−3.201	−1.966	down
207808_s_at	PROS1	−1.030	0.003	0.372	−3.420	−1.575	down
217979_at	TSPAN13	−1.018	0.002	0.363	−3.643	−1.174	down
204992_s_at	PFN2	1.026	0.005	0.393	3.151	−2.056	up
209230_s_at	NUPR1	−1.073	0.006	0.394	−3.085	−2.172	down
207526_s_at	IL1RL1	1.578	0.006	0.394	3.068	−2.202	up
212950_at	ADGRF5	−1.511	0.011	0.404	−2.803	−2.664	down
206710_s_at	EPB41L3	1.029	0.012	0.404	2.755	−2.746	up
202345_s_at	FABP5	−1.027	0.012	0.404	−2.747	−2.760	down
209277_at	TFPI2	−1.515	0.015	0.410	−2.658	−2.909	down
203980_at	FABP4	−1.740	0.018	0.424	−2.575	−3.049	down
202546_at	VAMP8	1.418	0.021	0.428	2.502	−3.170	up
219148_at	PBK	1.063	0.027	0.441	2.396	−3.343	up
206029_at	ANKRD1	1.131	0.029	0.447	2.356	−3.407	up
205680_at	MMP10	−1.153	0.030	0.447	−2.339	−3.434	down
204438_at	MRC1	−1.376	0.040	0.463	−2.201	−3.650	down
205110_s_at	FGF13	−1.013	0.049	0.480	−2.090	−3.819	down

## Data Availability

The GSE132651 dataset is openly available in the Gene Expression Omnibus database at https://www.ncbi.nlm.nih.gov/geo/query/acc.cgi?acc=GSE132651 accessed on 5 July 2022.
